# Insights into the Role of Plasma in Atmospheric Pressure Chemical Vapor Deposition of Titanium Dioxide Thin Films

**DOI:** 10.1038/s41598-018-35154-4

**Published:** 2018-11-12

**Authors:** Seongchan Kang, Rodolphe Mauchauffé, Yong Sung You, Se Youn Moon

**Affiliations:** 1 Department of Applied Plasma Engineering, Chonbuk National University, 567 Baekje-daero, Deokjin-gu, Jeonju-si, Jeollabuk-do 54896 Republic of Korea; 2 Department of Quantum System Engineering, Chonbuk National University, 567 Baekje-daero, Deokjin-gu, Jeonju-si, Jeollabuk-do 54896 Republic of Korea

## Abstract

In this work, the effect of plasma on the chemistry and morphology of coatings deposited by Atmospheric Pressure Plasma Enhanced Chemical Vapor Deposition (AP-PECVD) is investigated. To do so, plasma deposited amorphous titanium dioxide (TiO_2_) thin films are compared to thin films deposited using Atmospheric Pressure Chemical Vapor Deposition (AP-CVD) not involving the use of plasma. We focus here on the effect and the interest of plasma in the AP-PECVD process over AP-CVD for low substrate temperature deposition. The advantages of AP-PECVD over AP-CVD are often suggested in many articles however no direct evidence of the role of the plasma for TiO_2_ deposition at atmospheric pressure was reported. Hence, herein, the deposition via both methods is directly compared by depositing coatings with and without plasma using the same CVD reactor. Through the control of the plasma parameters, we are able to form low carbon coatings at low temperature with a deposition rate twice faster than AP-CVD, clearly showing the interest of plasma. Plasma enhanced methods are promising for the deposition of coatings at industrial scale over large surface and at high rate.

## Introduction

Nowadays, titanium dioxide (TiO_2_) is being intensively studied notably because of its chemical stability, optical and photocatalytic properties^1–8^. TiO_2_, especially under thin film form, was synthesized and demonstrated potential for applications such as anti-reflection coatings^1,2^, photocatalytic coatings for air and water purification^3,4^, self-cleaning surfaces^5^, anti-bacterial coatings^6,7^ and anti-corrosion thin films^8^. Various TiO_2_ thin film deposition methods have been studied and employed in literature^9–15^. While wet methods such as sol-gel^9^ are mainly involving time taking steps and generate wastes, the dry methods, *i*.*e*. Atomic Layer Deposition^10^, physical vapor deposition (PVD)^11^, plasma spraying^12^, Chemical Vapor Deposition (CVD)^13^ or low pressure Plasma-Enhanced CVD (PE-CVD)^14,15^ are one-step methods generating no or few wastes. Among the dry methods, CVD methods performed at low-pressure are widely investigated, however these techniques require high running cost because of vacuum systems and show relatively low deposition rates^16^.

Atmospheric Pressure Chemical Vapor Deposition (AP-CVD) methods were then developed to overcome such drawbacks, avoiding the use of vacuum systems, and provide a viable solution for industrial scale deposition. Atmospheric Pressure CVD, are successfully used to deposit TiO_2_ thin films^17^. By introducing the precursor under vapor form and by tuning the substrate temperature, it is possible to obtain TiO_2_ coating under different crystal phases such as amorphous, anatase or rutile^18^. Among the many reported works, some groups focus on the deposition of TiO_2_ thin films at room temperature in order to form good quality coatings for heat sensitive materials. Indeed, recently the open air dynamic deposition of amorphous coatings was performed via roll-to-roll systems using titanium isopropoxide (TTIP) and water vapor hydrolysis reaction at room temperature and atmospheric pressure^19,20^. AP-CVD methods enable the formation of good quality layers however they still present relatively low deposition rate (from 14 to 75 nm/min) and the heating of the substrate is needed to form anatase coatings, hence limiting the formation of anatase coatings on heat sensitive substrate like polymers^19,20^.

In order to reach high deposition rate and to form anatase at room temperature, Plasma Enhanced CVD methods are investigated in literature. Indeed, Atmospheric-Pressure Plasma Enhanced Chemical Vapor Deposition (AP-PECVD) has been reported as a promising method for TiO_2_ thin films deposition thanks to several advantages such as simple in-line implementation in roll-to-roll systems because of no vacuum requirement, high deposition rate and low temperature processing^3,15,21–31^. In order to avoid potentially dangerous precursors such as titanium tetrachloride TiCl_4_^22,23,31^, titanium-containing precursors such as titanium tetraisopropoxide (TTIP)^21,30^, titanium ethoxide (TEOT)^3^, or titanium bis-(acetylacetonate) diisopropoxide (TIPO)^29^ are currently favored. Among those precursors, TTIP remains the most studied precursor for AP-PECVD. However the deposition of dense and crystalline layers on sensitive substrate at low temperature from vaporized TTIP precursors remains challenging. Indeed, coatings are often formed of many powders and do not seem really adherent. High substrate temperature (from about 200 °C) or high plasma gas temperature (from 220 °C) are often needed to obtain anatase TiO_2_^21,24,26,28,29^. At processing and substrate temperature close to room temperature (<50 °C), the formation of powdery and amorphous coatings via AP-PECVD is more likely to occur^30,32^. The deposition rate of such amorphous TiO_2_ coatings via AP-PECVD at low temperature is often reported in literature to be in the range of AP-CVD of amorphous TiO_2_, *i*.*e*. 75 nm/min^26,30^. Deposition rate being an important parameter, especially for industrial deposition, hence, it raises the question of what is the effect of plasma and thus the interest of AP-PECVD over AP-CVD for the deposition of amorphous coatings. By looking at the current literature it is hard to answer to this question as no direct comparison has been undertaken using the same CVD reactor.

Therefore, in this work, AP-CVD and AP-PECVD are performed using the same set-up and precursor to deposit TiO_2_ thin films and investigate the difference between using and not using plasma during deposition. In a first part, the AP-CVD of TiO_2_ coatings from TTIP is investigated. The morphology and deposition rate of the coatings are determined by Scanning Electron Microscope (SEM). The chemical composition is obtained by X-ray photoelectron spectroscopy (XPS) and the crystallinity of the deposited coatings is assessed by Raman spectroscopy. In a second part, the effect of plasma on the morphology and deposition rate as well as on the chemical composition is investigated. The plasma gas composition effect on the coatings properties is also investigated.

## Results and Discussion

### Atmospheric pressure CVD of TiO_2_ thin film

**Figure 1:**
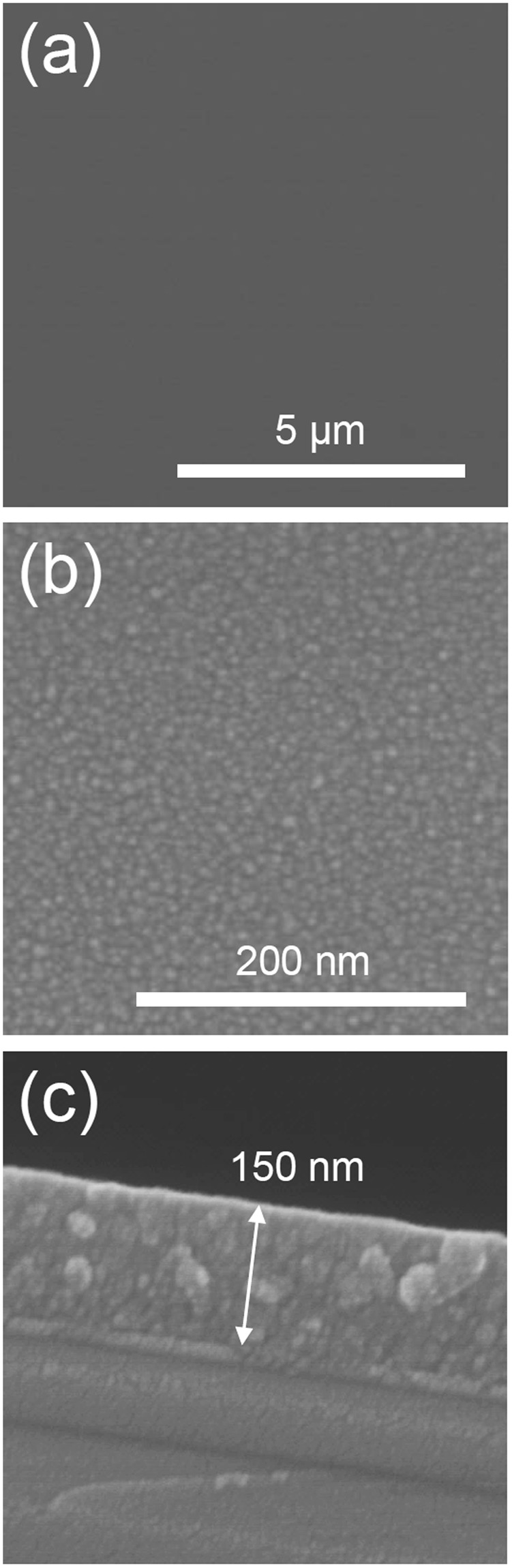
Scanning electron micrographs of the top view (**a,b**) and cross-sectional view (**c**) of an AP-CVD deposited thin film (200 passes under CVD head).

**Figure 2:**
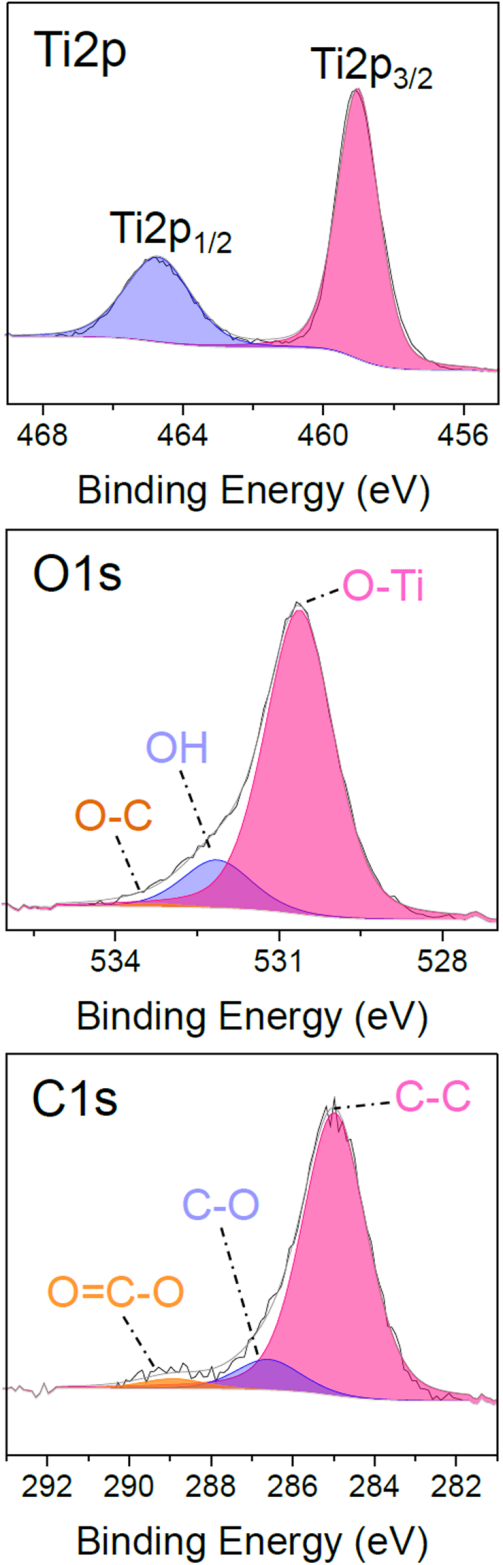
High resolution XPS core level fitting of Ti2p, O1s, and C1s peaks for as-deposited TiO_2_ thin films by AP-CVD.

The deposition is performed by AP-CVD without plasma generation at room temperature (25 °C) on silicon wafer for 200 passes at 5 mm/s. The deposited thin film is observed by SEM (Fig. 1) and appears smooth, well covering, pinhole-free and crack-free. At high magnification (Fig. 1b) the coating seems formed of nanoaggregates. The cross-sectional view shows that the coating seems made of aggregates forming a dense, well-adherent and non-porous coating on the surface. For 200 passes the thickness of the coating is about 150 nm, hence leading to a deposition rate of about 0.75 nm/pass. The chemical composition of the thin film is investigated by XPS. As reported in Table 1, adventitious carbon is present on the sample surface. By argon ion sputtering of the surface the surface contamination is removed and the bulk elemental composition is obtained. The deposited coating present a low carbon content as low as 7% with a O/Ti ratio of 2.1, close to stoichiometry. The high resolution spectra of Ti2p, O1s and C1s for as deposited coatings are presented in Fig. 2. The Ti2p core level spectra shows two components, Ti2p_3/2_ and Ti2p_1/2_, spaced 5.7 eV apart at 459 eV and 464.7 eV respectively, corresponding to Ti^4+^ state in TiO_2_ as reported in the literature^3^. The O1s peak comprises three contributions at 530.6, 532.1 and 533.3 eV. The major one at 530.6 eV is attributed to oxygen in the O-Ti bonds from TiO_2_, the second at 532.1 eV is attributed to OH and the peak at 533.3 eV corresponds to oxygen bonded to carbon, coming from precursor fragmentation and partly from the surface contamination^21,32^. The C1s spectra contributions are typical of adventitious carbon on the surface of non-argon sputtered samples. The high resolution spectra observations are performed on as-deposited coatings because of the effect of Ar^+^ etching, modifying the chemical environment of the element in TiO_2_ hence possibly leading to misconclusion. Indeed, as reported in Fig. S1, argon sputtering leads to the formation of the Ti^3+^ and Ti^2+^ peaks at lower binding energies due to oxygen removal^3^. O1s shows similar oxygen components compared to before surface cleaning. The C1s spectrum, which is now representative of the carbon environment in the layer, shows three contributions, the main one attributed to C-C bonds at about 284.8 eV, another one, very weak, at 286.6 eV, corresponding to C-O, and a last one at 282.7 eV. The latter peak may either be due to Ar bombardment leading to sputter damages in the layer or to interactions formed between carbon and titanium as suggested in the literature^33,34^.

**Table 1:** XPS chemical composition for TiO_2_ thin film deposited by AP-CVD.

Samples	Atomic composition (at.%)			
	Ti2p	O1 s	C1 s	O/Ti
As-deposited	20	50	30	2.5
After Ar sputtering	30	63	7	2.1

**Figure 3:**
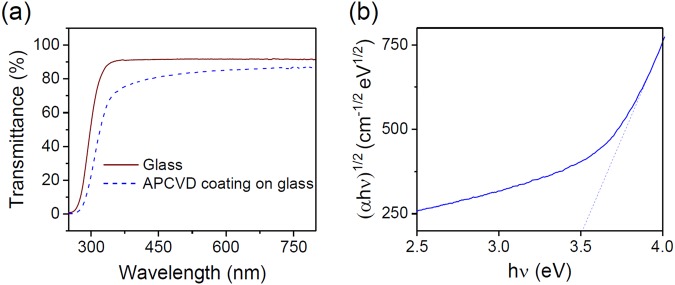
(**a**) UV-Visible transmittance spectra of glass and AP-CVD coating on glass. (**b**) Tauc plot for the calculation of the optical band gap of the AP-CVD coating.

Obviously, Raman spectroscopy analyses show that the deposited coatings are amorphous (data not shown) as reported for room temperature deposition of TiO_2_ coatings^19,20^. The deposited coatings are transparent and exhibit up to 80% of transmittance in the visible range when deposited on glass slides (Fig. 3a). The band gap of the amorphous coating, determined thanks to the Tauc plot method (Fig. 3b), is about 3.51 eV, in accordance with literature^19^.

The formation of TiO_2_ thin film is likely to occur via hydrolysis reaction of TTIP with water present in the surrounding atmosphere (Eq. (1)), forming TiO_2_ and volatile isopropyl alcohol as a by-product, explaining the low carbon content in the deposited coating^35^.

1 $${\rm{Ti}}{({{\rm{OC}}}_{3}{{\rm{H}}}_{7})}_{4}+2{{\rm{H}}}_{2}{\rm{O}}\to {{\rm{TiO}}}_{2}+4{{\rm{C}}}_{3}{{\rm{H}}}_{7}{\rm{OH}}$$

The reaction is likely to occur both at the substrate interface and in the gas phase, explaining the dense layer composed of aggregates observed by SEM. However at atmospheric pressure, part of the precursor is believed to be lost in the gas flow as a coating is observed on the sides of the CVD source and thus do not reach the sample surface to take part in the coating formation. Hence, the use of plasma to enhance the fragmentation of the precursor is then believed to increase the deposition rate.

### Atmospheric pressure plasma enhanced CVD (AP-PECVD) of TiO_2_ thin film

**Figure 4:**
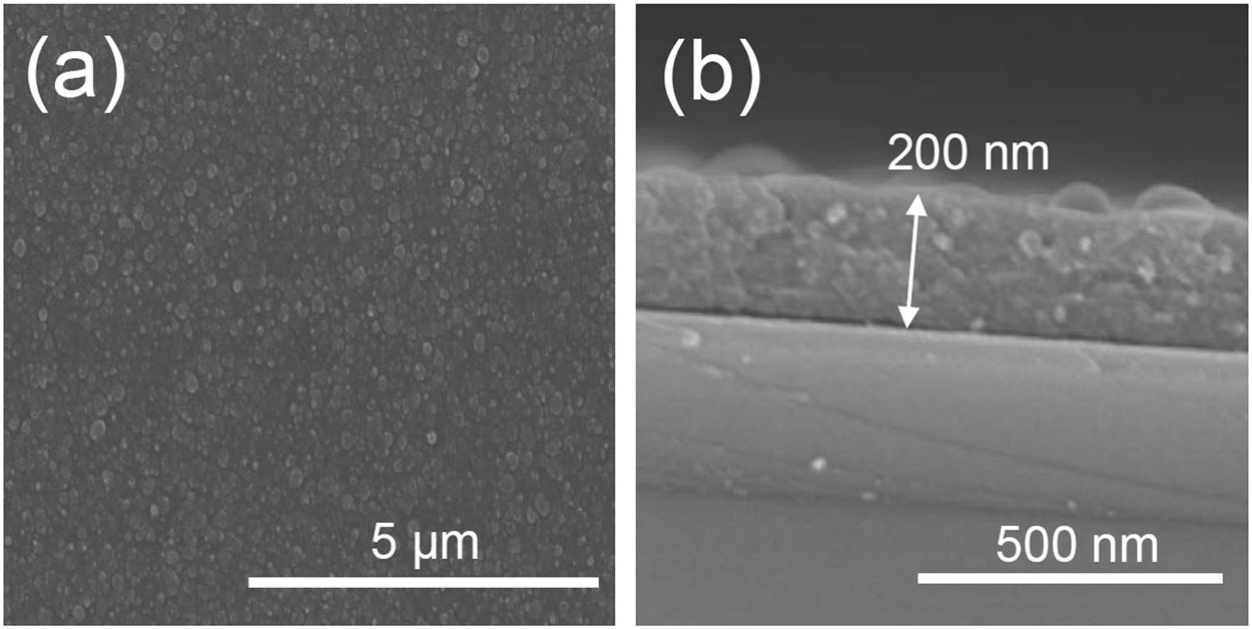
Scanning electron micrographs of the top view (**a**) and cross-sectional view (**b**) of an AP-PECVD deposited thin film using a 70 W He/TTIP discharge (90 passes).

The AP-PECVD of TTIP is carried out. In order to only study the effect of the plasma compared to the AP-CVD set-up and avoid any thermal effect, low power is used, *i*.*e*. 70 W. IR thermometer assesses that the substrate temperature (about 30 °C) remains close to the room temperature (25 °C). Figure 4a shows the surface morphology of a coating deposited on silicon wafer by AP-PECVD using a He/TTIP discharge at 70 W. The surface is relatively smooth but shows uniformly dispersed nano-agglomerates as often observed in coatings deposited by AP-PECVD in the literature, due to gas phase reactions^3,23,24,26,29,30^. In the cross-sectional micrographs (Fig. 4b) we can notice that the coating is composed of many agglomerates forming a dense coating. The film thickness for a 90 passes deposition is 200 nm, *i*.*e*. 2.2 nm/pass, which is about three times higher than the AP-CVD case.

**Figure 5:**
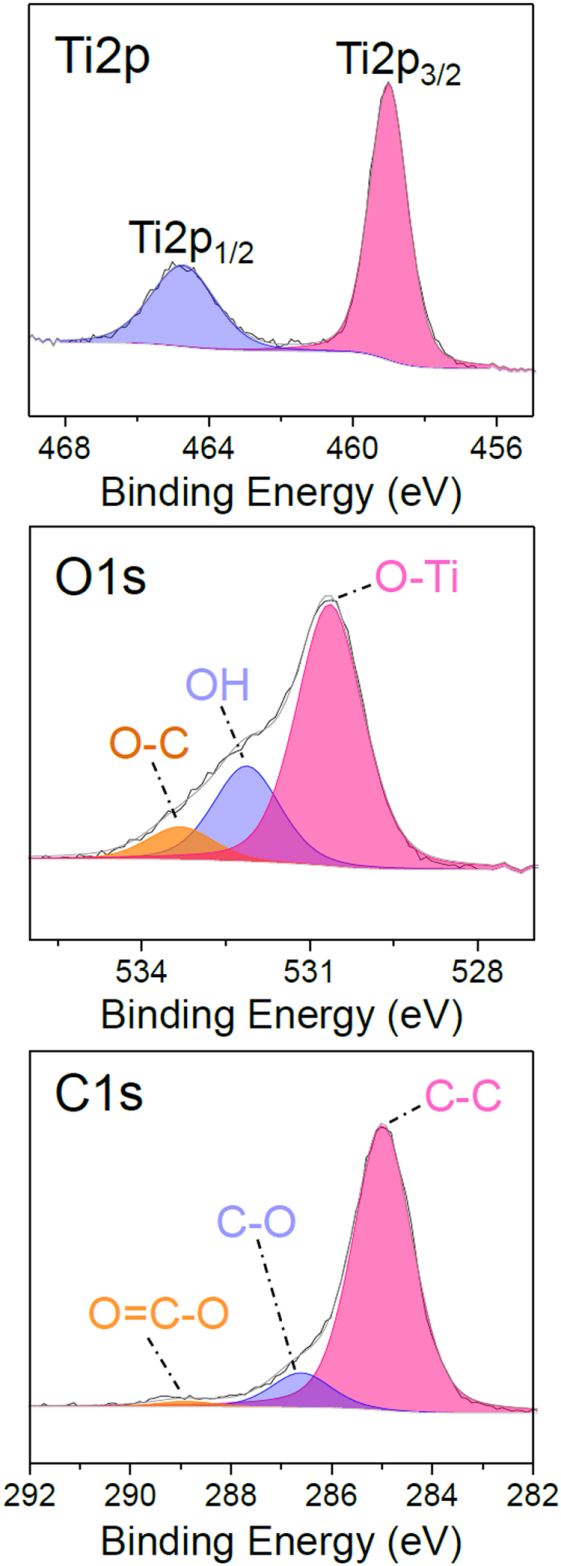
High resolution XPS core level fitting of Ti2p, O1s, and C1s peaks for as-deposited TiO_2_ thin films using a 70 W He/TTIP discharge.

**Figure 6:**
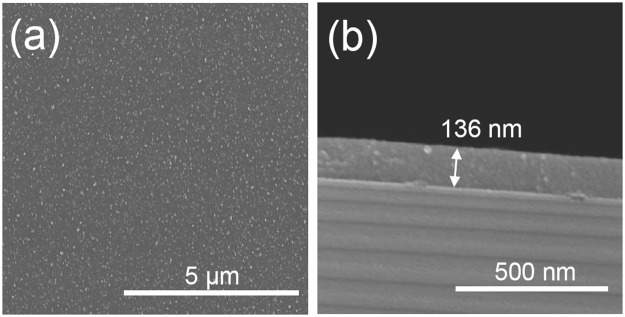
Scanning electron micrographs of the top view (**a**) and cross-section (**b**) of AP-PECVD deposited coating using a 70 W He/O_2_/TTIP discharge (90 passes).

Looking at the coating chemical composition, in Table 2, a significant amount of carbon (32 at.%) is still found in the layer bulk after removal of the surface adventitious carbon. The O/Ti ratio is 2.09. Even though carbon is present in high amount, the high resolution core level spectra of Ti2p and O1s in Fig. 5 clearly shows the presence of TiO_2_ phase in the as-deposited layers. Indeed, we can notice the presence of two components, namely Ti2p_3/2_ and Ti2p_1/2_ at 459 eV and 464.7 eV respectively, spaced 5.7 eV apart, identified as Ti^4+^ in TiO_2_^3,36^. The O1s peak comprises the same three contributions than in AP-CVD deposited case, corresponding respectively to O-Ti, OH and to oxygen bonded to carbon. As reported for Ar ion etched surface in AP-CVD the surface cleaning by etching leads to the appearance of Ti^3+^ and Ti^2+^ peaks due to oxygen removal (Fig. S2a)^3^. After etching the O1s main peak remains O-Ti and the C1s peak is still well observed and shows C-C and C-O peaks as well as the previously discussed contribution at 282.7 eV. The high carbon content observed by XPS is then likely to come from the fragmented precursor and not from surface contamination as argon etching is performed and believed to have it removed. The high deposition rate of the layer is then believed to be due to the presence of high amount of carbon in the coating. Indeed, this is indirectly proved by annealing the coating at 450 °C during 2 h at air. The carbon content is drastically decreased by thermal oxidation (as low as 13 at.%) (Table 3 and Fig. S2c) while the coating thickness is reduced as low as 126 nm (Fig. S3). Such high carbon content is believed to be due to the high fragmentation of the precursor in the plasma, leading to the formation of various carbon species in the reactive gas phase which are subsequently deposited and not eliminated in the gas flow. The presence of high carbon concentration in layers deposited from TTIP is also reported in the literature. Maurau *et al*. reported the presence of high carbon concentration in layers deposited using a plasma torch using TTIP and N_2_ plasma^37^. They also suggest the high degree of fragmentation in the plasma and show using mass spectroscopy that aromatic carbon compounds are present in their layer. In order to control the carbon concentration they then recommend the introduction of oxygen. Upon introduction of 0.005 SLM (0.05%) of oxygen in our low power discharge in order to control the carbon concentration, coatings are also successfully deposited. During deposition IR thermometer measurements show that the surface remains at low temperature (30 °C). SEM observations (Fig. 6) shows that the surface of the coatings are smooth, covering and crack-free and present agglomerates slightly smaller on the surface than without oxygen introduction. The cross-section reveals the dense morphology of the layer. It is worth noticing that upon introduction of oxygen the thickness of the layer is reduced. Indeed, for 90 passes, a 136 nm thick coating is deposited, *i*.*e*. 1.5 nm/pass, remaining twice faster than AP-CVD.

**Table 2:** XPS chemical composition after Ar^+^ sputtering for TiO_2_ thin films deposited by AP-PECVD using different experimental conditions.

Experimental cases	Atomic composition (at%)			
	Ti2p	O1s	C1s	O/Ti
70 W He/TTIP as-deposited	22	46	32	2.09
70 W He/O_2_/TTIP as-deposited	30	61	9	2.03

**Table 3:** XPS chemical composition after Ar^+^ sputtering for annealed TiO_2_ thin films deposited by AP-PECVD.

Experimental cases	Atomic composition (at%)			
	Ti2p	O1s	C1s	O/Ti
70 W He/TTIP annealed 2 h at 450 °C	27	60	13	2.22
70 W He/O_2_/TTIP annealed 2 h at 450 °C	30	66	4	2.2

**Figure 7:**
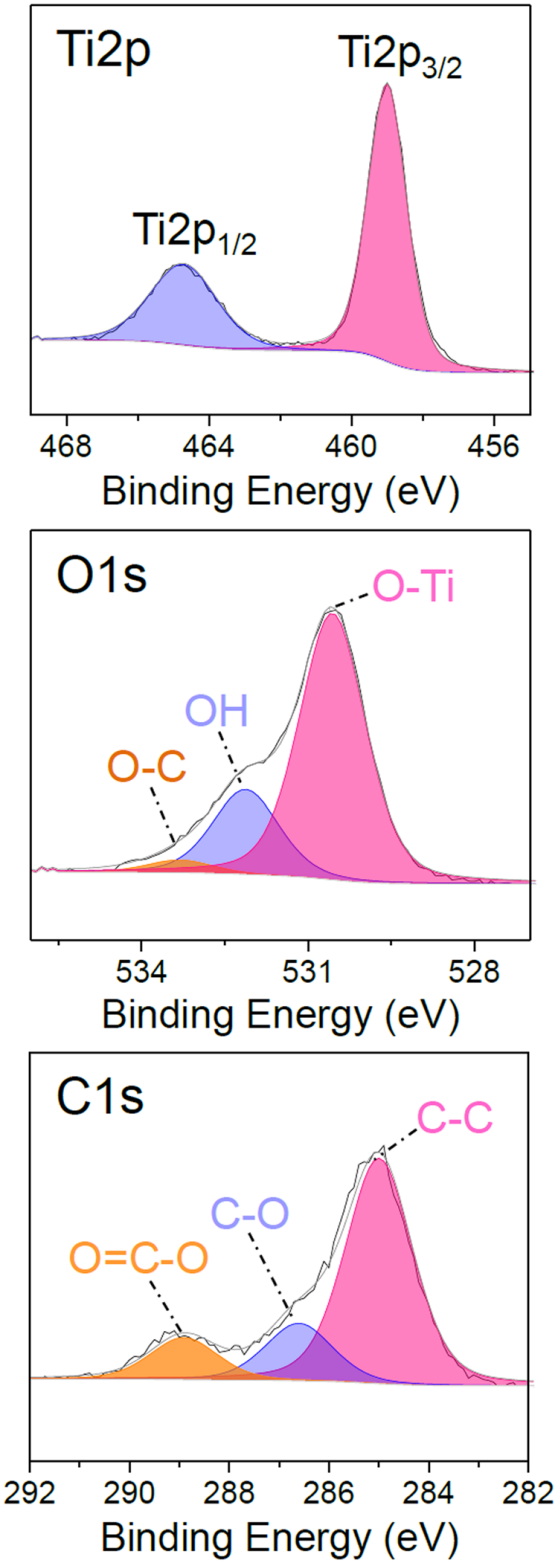
High resolution XPS core level fitting of Ti2p, O1s, and C1s peaks for as-deposited TiO_2_ thin films using a 70 W He/O_2_/TTIP discharge.

A significant decrease of the carbon content in the coating bulk is measured by XPS (Table 2). The atomic concentration of carbon is found as low as 9% for as-deposited thin films with a 2.03 O/Ti ratio. Even though the elemental quantification shows significant discrepancies with thin film deposited with a He/TTIP discharge, the analysis of the elements environments in Fig. 7 shows many similarities. Indeed, the presence of TiO_2_ phase in the thin films is assessed, with, as previously reported, the presence of Ti2p_1/2_ and Ti2p _3/2_ peaks at 459 eV and 464.7 eV, fingerprint of Ti^4+^ in TiO_2_^3^. No significant differences are observed for O1s compared to a He/TTIP discharge deposited coating. C1s shows the presence of carbon on the surface, however after surface etching with argon sputtering only a noisy and weak carbon peak is observed (Fig. S2c), clearly showing the low carbon content in the thin films upon injection of oxygen. With a 1.5 nm/pass deposition rate, corresponding to about 91 nm/min by dividing the thickness by the total plasma exposure time during deposition (*i*.*e*. 90 s) our method lies among the fastest CVD methods for TiO_2_ thin films formation at low substrate temperature^13–15,26^. The effect and the interest of addition of oxygen in the plasma is then observed. In order to investigate the origin of the differences in the composition of thin films deposited from He/TTIP and He/O_2_/TTIP, optical emission spectroscopy is carried out on the discharges.

**Figure 8:**
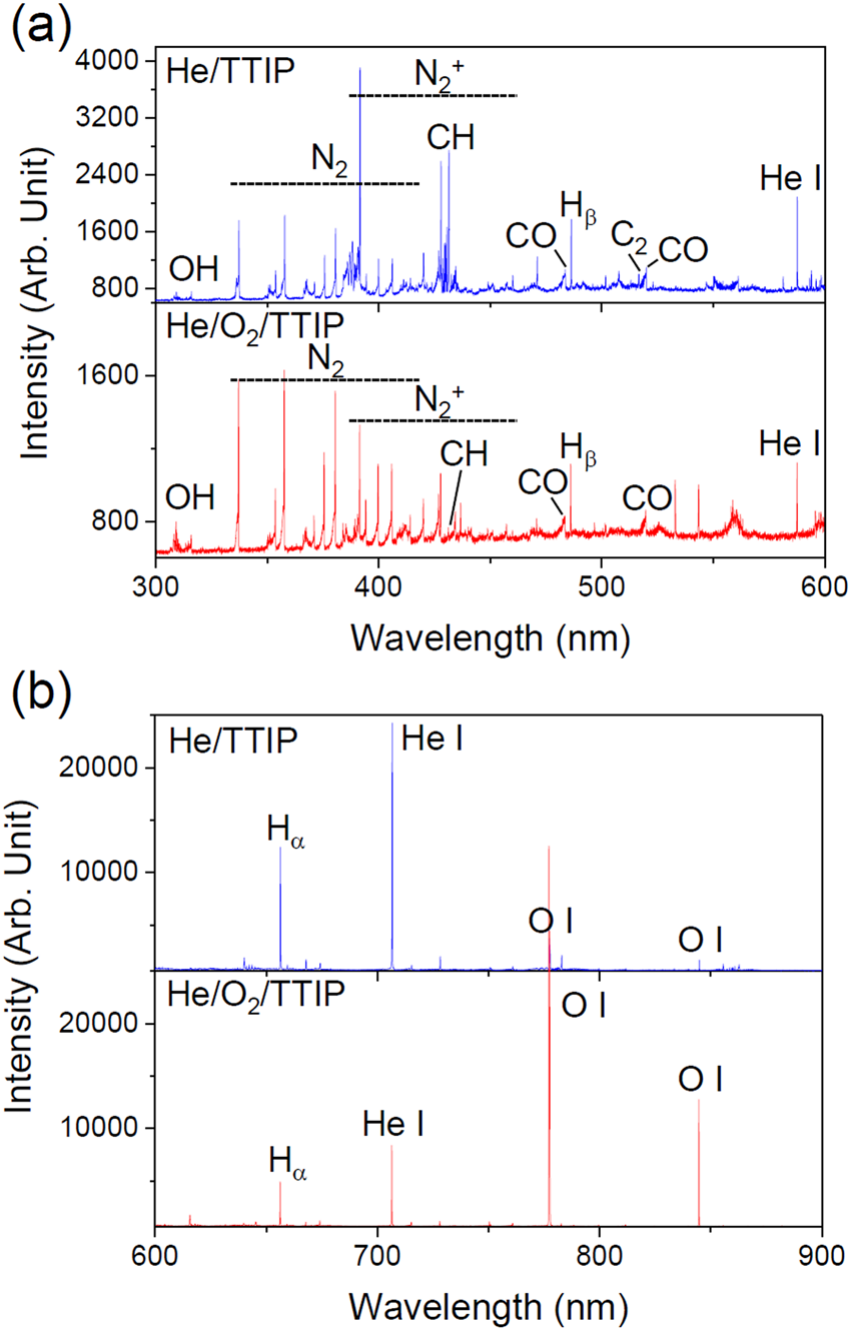
Optical emission spectra of He/TTIP and He/O_2_/TTIP discharges at 70 W in the 300–600 nm (**a**) and 600–900 nm (**b**) wavelength range.

**Figure 9:**
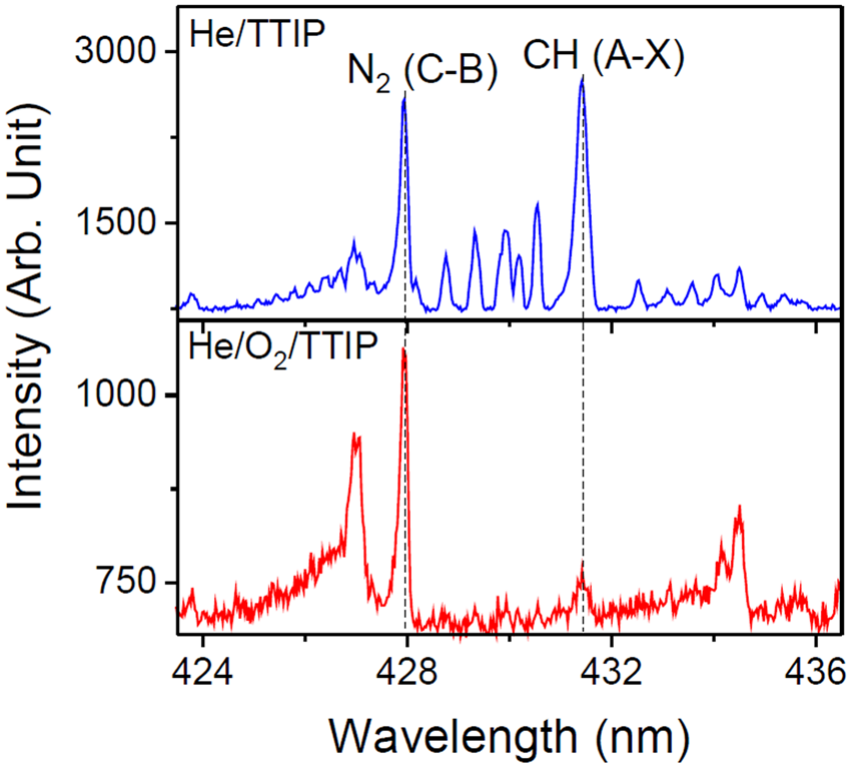
OES spectra in the 424–436 nm wavelength region for a 70 W He/TTIP discharge and a 70 W He/O_2_/TTIP discharge.

While both discharges have similar gas temperature (30 °C), as determined by comparing the measured molecular N_2_ SPS (0–2) peak spectrum with a simulated one (Fig. S4), some differences in the plasma chemistry are observed. Figure 8 shows the plasma emission spectra of He/TTIP and He/O_2_/TTIP discharges in the wavelength regions between 300 and 600 nm and between 600 and 900 nm (Fig. 8a,b). Both discharge spectra display peaks originating from the feeding gas and from ambient air (due to the open-air configuration): helium atomic lines (501.5 nm, 587.5 nm, 667.8 nm, 706.5 nm), N_2_ second positive system (N_2_ SPS, C^3^Π_u_ − B^3^Π_g_), N_2_^+^ first negative system (N_2_ FNS, B^2^ ${{\rm{\Sigma }}}_{u}^{+}$ − X^2^ ${{\rm{\Sigma }}}_{g}^{+}$), OH (A^2^Σ^+^ − X^2^Π), hydrogen atomic lines (H_β_ 486.1 nm, H_α_ 656.3 nm) and oxygen atomic lines (777 nm, 844 nm)^38,39^. Some carbon containing species likely to result from the fragmentation of TTIP, as being the only carbon source, are also observable: C_2_ (Swan band, d^3^Π_g_ − a^3^Π_u_), CO (Ångström system, B^1^Σ − A^1^Π) and CH (4300 Å system, A^2^Δ − X^2^Π)^38^. While for a He/TTIP discharge spectrum the CH molecular band around 431 nm is intense, upon introduction of oxygen the CH peak intensity is drastically decreased (Fig. 9). This decrease could be explained because of the reaction of the formed carbon-containing species in the discharge with the reactive oxygen species present in the plasma to form volatile compounds such as CO_2_ or CO, which can be eliminated in the gas flow. The low carbon concentration in the deposited thin film is then believed to be due to the decrease of carbon-containing species in the discharge.

**Figure 10:**
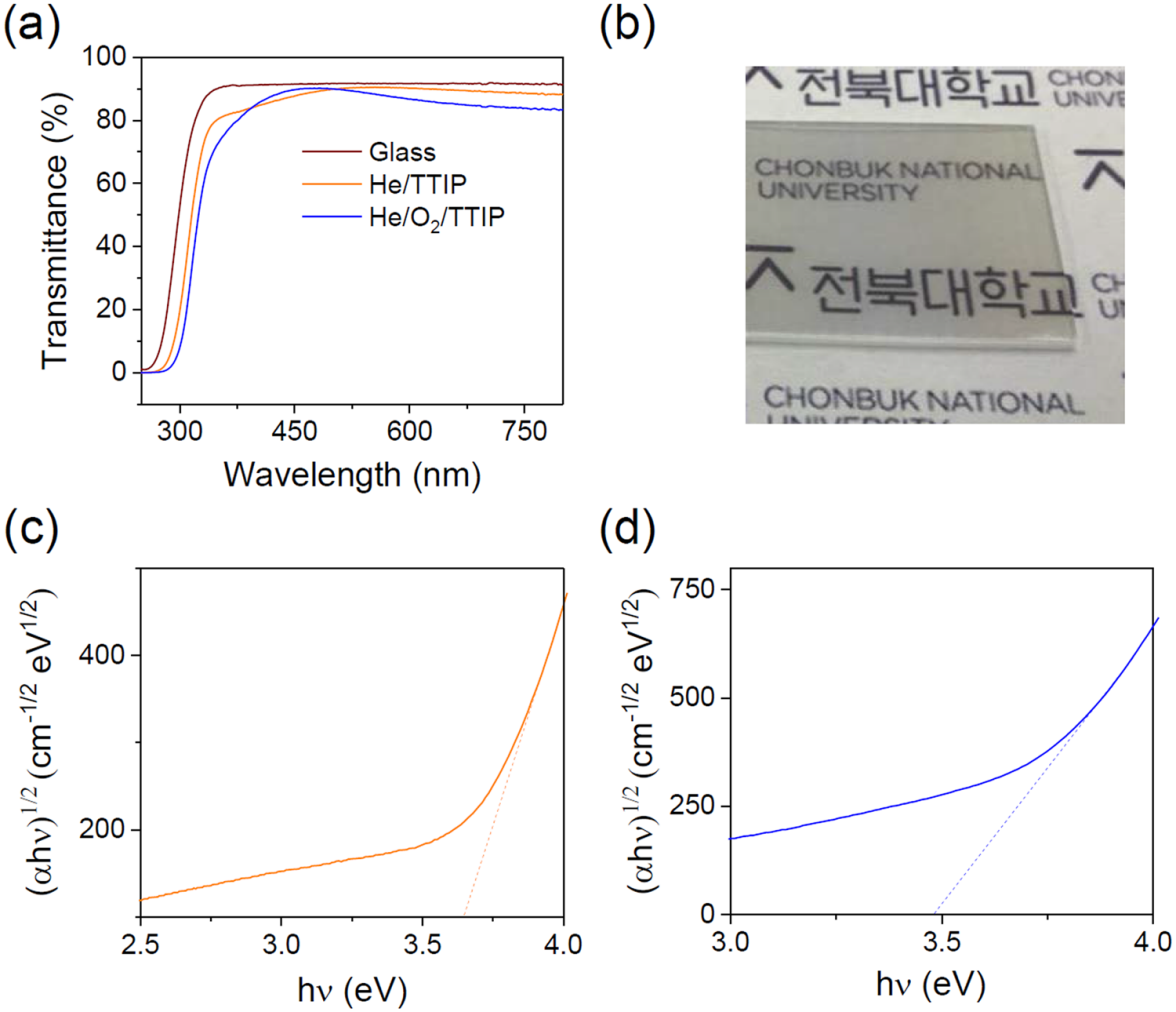
(**a**) UV-visible transmittance spectra of bare glass and He/TTIP and He/O_2_/TTIP thin films deposited on glass. (**b**) Picture of a He/O_2_/TTIP deposited thin film on a glass slide. Tauc plot for the calculation of the optical band gap of thin films deposited on glass slide using He/TTIP (**c**) and He/O_2_/TTIP (**d**) discharges.

The crystallinity and the optical properties of the obtained AP-PECVD thin films are investigated. TiO_2_ thin films grown on Si substrate, using He/TTIP and He/O_2_/TTIP discharges, are amorphous, no crystalline phase related peaks are present in the Raman spectra (data not shown) for both cases. The optical properties of the amorphous thin films deposited on glass are also investigated. Figure 10a shows the transmittance spectra of bare glass and as-deposited thin films in the 250–800 nm wavelength region. The coatings are transparent as depicted in Fig. 10b, and show a transmittance of minimum 80% in the visible range while coated on glass. Band gaps are determined by the extrapolation of the linear part of the (α*h*ν)^1/2^ versus photon energy as reported in Fig. 10c,d. For TiO_2_ films deposited using He/TTIP the band gap is 3.64 eV while with He/O_2_/TTIP, the band gap is about 3.48 eV. Those two band gaps values remain close and are in accordance with values from literature for amorphous TiO_2_^19^.

It is worth noticing that for applications requiring lower carbon concentration, providing the substrate is not heat sensitive, the annealing of the coating at 450 °C during 2 h allows decreasing the carbon concentration down to 4% (Table 3 and Fig. S2d). Upon annealing the thickness of the He/O_2_/TTIP deposited coating is decreased from 136 nm to 100 nm (Fig. S3). Even though both AP-CVD and AP-PECVD allows obtaining low carbon and dense amorphous TiO_2_ coatings with a system which is easily up-scalable for large area deposition, AP-PECVD, by its twice faster deposition rate provides an interesting deposition alternative for industrial deposition. The AP-PECVD on large surface at high roll-to-roll speed is however currently limited, first due to the deposition rate and also due to the source size. It is worth noticing that currently in literature, few works report the use of large plasma source for large area TiO_2_ deposition on roll-to-roll configuration. In this work, the developed roll-to-roll system can be easily modified and the 10 cm long electrode can be simply replaced by a longer electrode to coat large surfaces homogeneously. The deposition at high rate and low temperature on large area of anatase phase TiO_2_ with high photocatalytic properties remains however a challenge. The tuning of the plasma deposition parameters such as plasma pulse are believed to be key parameters to reach such objectives and will be the object of further study.

## Conclusion

In this study the deposition of amorphous TiO_2_ coatings both via AP-CVD and AP-PECVD using TTIP is investigated. While AP-CVD appears as an easy method for roll-to-roll deposition of dense and low carbon-containing coatings at room temperature, the method is limited by its relatively low deposition rate. Using the same reactor, AP-PECVD is performed, thanks to the highly reactive species present in the plasma, TTIP is fragmented and high deposition rates are observed. Indeed, deposition rate twice faster than AP-CVD is reached at room temperature. Low carbon coatings are successfully deposited with carbon content as low as 9% for as-deposited coating for a He/O_2_/TTIP discharge at 70 W. The introduction of oxygen is shown to be a mandatory key parameter to obtain low carbon coatings. Indeed, He/TTIP discharge at 70 W leads to the formation of coatings containing up to 30% of carbon. The reactive oxygen species in the discharge are likely to react with the formed carbon species and reduce the deposited carbon as suggested by optical emission spectroscopy showing lower carbon species in the discharge upon introduction of oxygen. Hence, AP-PECVD, via simple tuning of the deposition parameters provides a fast deposition method and is a promising pathway to deposit high quality metal oxides coatings at low temperature on sensitive substrates.

## Methods

**Figure 11:**
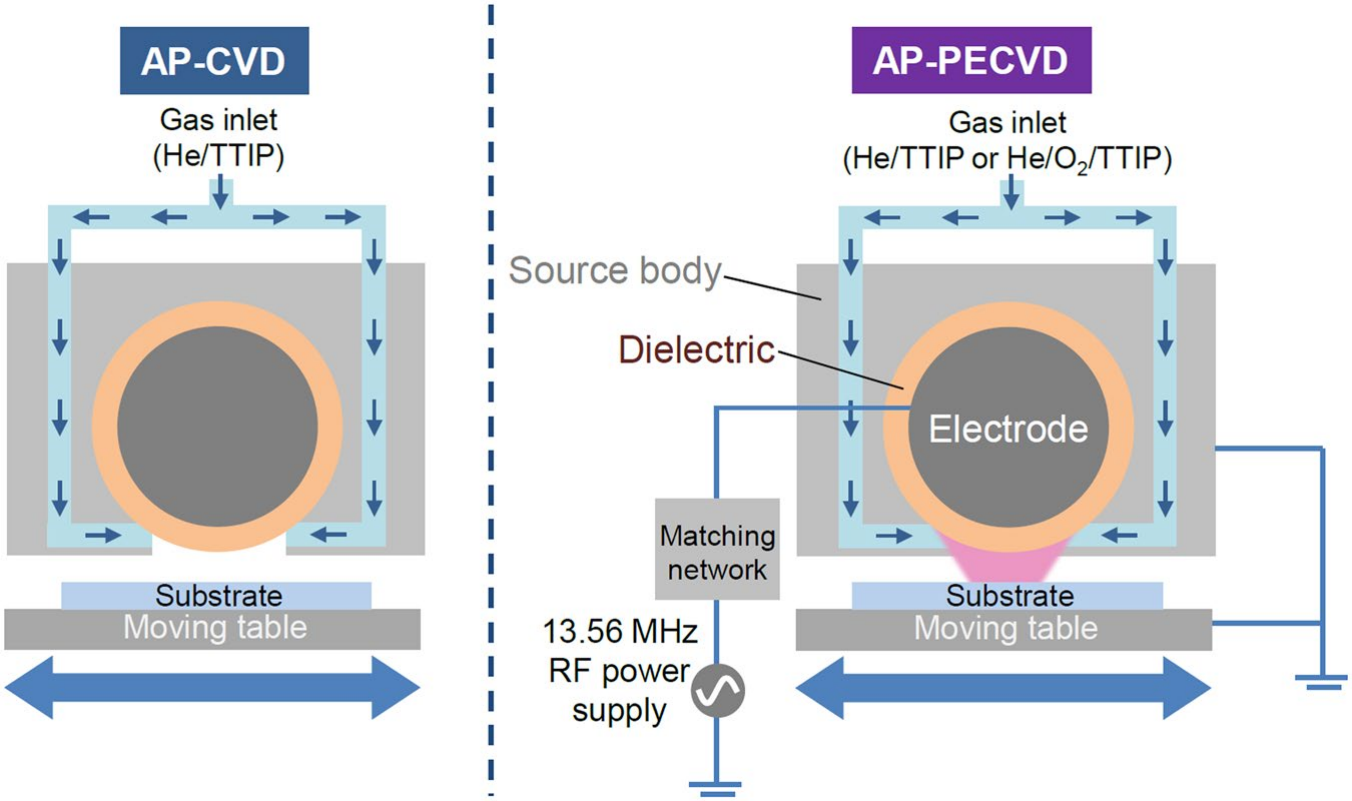
Scheme of the CVD reactor in AP-CVD (left) and AP-PECVD configuration (right).

Atmospheric pressure chemical vapor deposition (AP-CVD) The thin film deposition is performed in an open-air reactor as depicted in Fig. 11. The precursor, *i*.*e*. titanium isopropoxide (TTIP, 99.9% 5N trace metal basis, Sigma-Aldrich), is vaporized using a heated bubbler in a water bath (SH-WB-13CDR SAMHEUNG ENERGY) at 70 °C. The vapors are carried to the deposition area via heated stainless steel gas tubes (80 °C). Mass flow controllers (MFC, Linetech) are used to control the gases flow rates: the helium gas flow in the bubbler (carrier gas) is set to 1 standard liter per minute (SLM) and the helium dilution gas is set to 9 SLM. The gap between the CVD head and the substrate is set to 1 mm. The CVD head is thermoregulated by water cooling and kept at room temperature. To ensure reproducibility, during experiments (both for AP-CVD and AP-PECVD), the surrounding temperature (about 25 °C) and relative humidity (about 50%) of the laboratory are controlled using a thermo-hygrostat system (Shinsung Engineering, Zephyrus SCA-A010WT1). To deposit large-area and uniform TiO_2_ films, the substrate is moved back and forth at 5 mm/s under the CVD shower head. Coatings are deposited on silicon wafer and glass slide. The AP-CVD parameters are summarized in Table 4.

**Table 4:** Experimental parameters for TiO_2_ thin film layer formation.

Fixed parameters (AP-CVD and AP-PECVD)	
Carrier gas flow rate (TTIP in bubbler)	1 SLM of helium
Main gas flow rate	9 SLM of helium
Moving stage speed	5 mm/s
Gap	1 mm
Bubbler temperature (Water bath)	70 °C
**AP-CVD parameters**	
Deposition passes	200 passes
**AP-PECVD parameters**	
Deposition passes	90 passes
Discharge power	70 W
Addition of O_2_ in the discharge	0 or 0.005 SLM

### Atmospheric Pressure Plasma Enhanced Chemical Vapor Deposition (AP-PECVD)

Atmospheric-pressure plasma thin film deposition is performed using the same system as for AP-CVD. In this case, as depicted in Fig. 11, the center electrode (1 cm diameter and 10 cm long) covered by an alumina tube is powered by a 13.56 MHz Radio Frequency (RF) power supply (Youngsin Eng., YSE-12EH) to ignite the discharge. In this work, the RF discharge power is fixed at 70 W and tuned using an impedance matching network to minimize the reflected power under 1%. To generate a stable glow discharge at atmospheric pressure, helium gas is chosen as plasma gas^40^. As for AP-CVD, the helium carrier gas flow rate is set to 1 SLM and the dilution gas flow rate is 9 SLM. To study the effect of oxygen during deposition, 0.005 SLM of O_2_ is introduced in the discharge. The deposition is performed dynamically thanks to the moving table at 5 mm/s. The discharge gap is fixed as 1 mm. The AP-PECVD parameters are detailed in Table 4. Coatings are deposited on silicon wafer and glass slide. The plasma diagnostic is performed by Optical Emission Spectroscopy (OES) using a SCT-320 Princeton instruments apparatus (1800 grooves/mm, 500 nm blazed angle) equipped with a charge coupled device (CCD, PIXIS400B Princeton Instrument). During the deposition process, the plasma gas temperature is estimated from the rotational temperature measured by fitting of the experimentally obtained OH, N_2_ (Second Positive System), and CH rotational spectra with theoretically calculated synthetic spectra^41,42^. The gas temperature is found to be as low as 30 °C as seen in Fig. S4. The substrate temperature is also monitored during deposition using an IR thermometer (Fluke 62 MAX+). The substrate temperature during deposition is about 30 °C. The top electrode is water cooled in order to keep it at low temperature.

### Thin film characterization

The morphology and thickness of titanium dioxide thin films are analyzed using a Field Emission Scanning Electron Microscope (FE-SEM, Hitachi SU-8030). Before SEM observations, samples are metalized with platinum via an ion sputter coater (Hitachi MC1000) to reduce charging artefacts. X-ray photoelectron spectroscopy (XPS) analyses are performed on samples using a Thermo Fisher Scientific K-Alpha instrument having a monochromatic A1 Kα X-ray source (1486.6 eV). Argon sputtering is conducted for 30 s at l kV to remove the surface contamination and investigate bulk chemical composition. For as-deposited samples analysis with carbon contamination on their surface, charge referencing was performed using adventitious carbon as a reference. The C-C binding energy of the carbon C1s is set at 285 eV. For etched samples, with no adventitious carbon, referencing is performed by calibrating the Ti^4+^ component at 459 eV which is proved to be present in all as-deposited samples. Crystallinity is assessed via Raman spectroscopy (Tokyo Instruments Nanofinder 30), the spectra are recorded between 100 and 800 cm^−1^ using a 532 nm laser excitation wavelength. A UV-vis spectroscope (UV-2700 Shimadzu) equipped with an integrating sphere is used for transmittance and absorbance measurement. The Tauc method is used to determine the band gap energy of the deposited materials, the quantity (αhν)^1/2^, where α is the absorption coefficient, is plotted versus the energy (hν).

## Electronic supplementary material

Supporting information

## Data Availability

The authors declare that the data supporting the findings of this work are available within the paper. All additional raw and derived data that support the plots within the paper and other findings of this work are available from the corresponding author upon reasonable request.
