# Supplementary material for: Insights into the Role of Plasma in Atmospheric Pressure Chemical Vapor Deposition of Titanium Dioxide Thin Films
Source: Sci Rep. 2018 Nov 12;8:16684. doi: 10.1038/s41598-018-35154-4 (PMC6232178; doi:10.1038/s41598-018-35154-4)
Supplement: Supplementary file 1 — Supporting information [file 41598_2018_35154_MOESM1_ESM.docx]

**Supporting information**

**Insights into the Role of Plasma in Atmospheric Pressure Chemical Vapor Deposition of Titanium Dioxide Thin Films**

Seongchan Kang^1,^^[[1]](#footnote-1)^, Rodolphe Mauchauffé^2,*^, Yong Sung You^2^, Se Youn Moon^1,2^^[[2]](#footnote-2)^

*^1^Department of Applied Plasma Engineering, Chonbuk National University, 567 Baekje-daero, Deokjin-gu, Jeonju-si, Jeollabuk-do, 54896, Republic of Korea*

*^2^Department of Quantum System Engineering, Chonbuk National University, 567 Baekje-daero, Deokjin-gu, Jeonju-si, Jeollabuk-do, 54896, Republic of Korea*

**Figure S1.** High resolution XPS core level fitting of Ti2p, O1s, and C1s peaks for AP-CVD thin film after surface etching with argon ions in order to remove surface adventitious carbon.

**Figure S2.** High resolution XPS core level fitting of Ti2p, O1s, and C1s peaks for AP-PECVD coatings deposited using (a) He/TTIP and (b) He/O_2_/TTIP discharges after surface etching with argon ions in order to remove surface adventitious carbon. (c) and (d) are respectively spectra of coatings deposited with He/TTIP and He/O_2_/TTIP discharges subsequently annealed at 450°C during 2h then surface etched with Ar^+^ to remove carbon surface contamination.

**Figure S3.** SEM pictures of AP-PECVD coatings formed with (a) He/TTIP and (b) He/O_2_/TTIP discharges after annealing at 450°C during 2h.


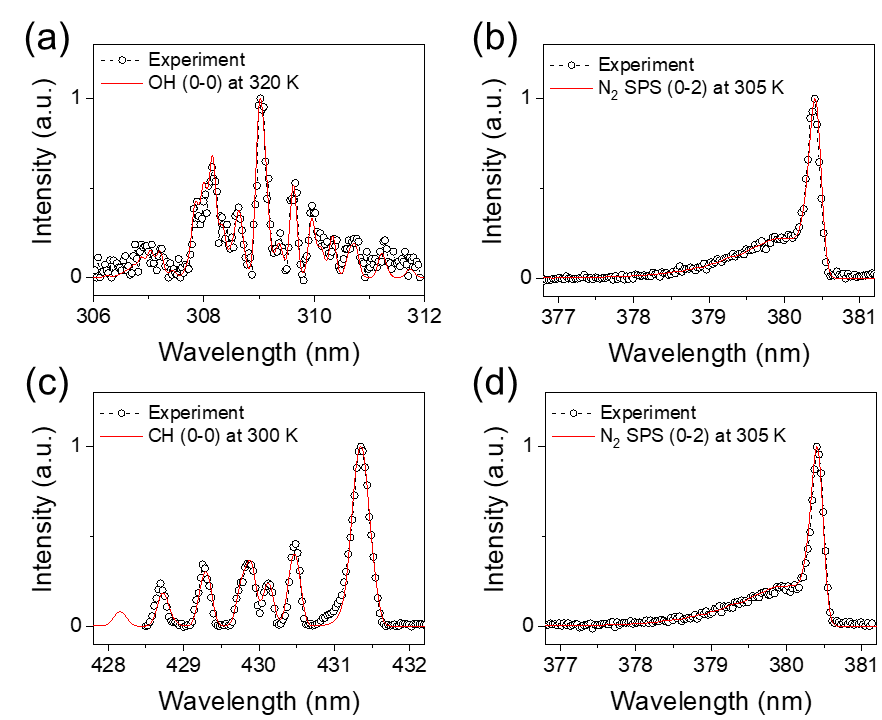


**Figure S4.** Plasma gas temperature is estimated by rotational temperature measurement through the comparison between experimental and theoretical spectrum of (a) OH (A^2^Σ^+^ - X^2^Π, 0-0), (b) N_2_ second positive system (SPS, C^3^Π_u_ – B^3^Π_g_, 0-2) and (c) CH (A^2^Δ – X^2^Π, 0-0) diatomic molecular spectra with best fitting condition. (a)-(c) are the spectra of He/TTIP plasma and (d) is the N_2_ SPS spectrum of He/O_2_/TTIP plasma.

1. These authors contributed equally to this work. [↑](#footnote-ref-1)
2. Author to whom correspondence should be addressed: E-mail: symoon@jbnu.ac.kr [↑](#footnote-ref-2)
